# Solid formulation of a supersaturable self-microemulsifying drug delivery system for valsartan with improved dissolution and bioavailability

**DOI:** 10.18632/oncotarget.21691

**Published:** 2017-10-09

**Authors:** Dong Woo Yeom, Bo Ram Chae, Jin Han Kim, Jun Soo Chae, Dong Jun Shin, Chang Hyun Kim, Sung Rae Kim, Ji Ho Choi, Seh Hyon Song, Dongho Oh, Se Il Sohn, Young Wook Choi

**Affiliations:** ^1^ College of Pharmacy, Chung-Ang University, Seoul 06974, Republic of Korea; ^2^ Daewon Pharm. Co., Ltd, Seoul 04994, Republic of Korea

**Keywords:** valsartan, SMEDDS, solid carrier, tablet, optimization

## Abstract

In order to improve the dissolution and oral bioavailability of valsartan (VST), and reduce the required volume for treatment, we previously formulated a supersaturable self-microemulsifying drug delivery system (SuSMEDDS) composed of VST (80 mg), Capmul^®^ MCM (13.2 mg), Tween^®^ 80 (59.2 mg), Transcutol^®^ P (59.2 mg), and Poloxamer 407 (13.2 mg). In the present study, by using Florite^®^ PS-10 (119.1 mg) and Vivapur^®^ 105 (105.6 mg) as solid carriers, VST-loaded solidified SuSMEDDS (S-SuSMEDDS) granules were successfully developed, which possessed good flow properties and rapid drug dissolution. By introducing croscarmellose sodium (31 mg) as a superdisintegrant, S-SuSMEDDS tablets were also successfully formulated, which showed fast disintegration and high dissolution efficiency. Preparation of granules and tablets was successfully optimized using D-optimal mixture design and 3-level factorial design, respectively, resulting in percentage prediction errors of <10%. In pharmacokinetic studies in rats, the relative bioavailability of the optimized granules was 107% and 222% of values obtained for SuSMEDDS and Diovan^®^ powder, respectively. Therefore, we conclude that novel S-SuSMEDDS formulations offer great potential for developing solid dosage forms of a liquefied formulation such as SuSMEDDS, while improving oral absorption of drugs with poor water solubility.

## INTRODUCTION

Use of a self-microemulsifying drug delivery system (SMEDDS) is widely known as one of the most effective approaches to overcome problems associated with low solubility and poor oral absorption of water-insoluble drugs. Previously, we have demonstrated that a supersaturable SMEDDS (SuSMEDDS) greatly contributed to enhanced dissolution and oral absorption of valsartan (VST), a drug with poor solubility in water [1]. However, the liquid state of both SMEDDS and SuSMEDDS causes several limitations for practical manufacturing development and clinical application. Since the liquid formulation is generally required to be enclosed in soft gelatin capsules, problems are frequently evoked, including high manufacturing costs, pharmaceutical incompatibility, drug leakage or precipitation, and capsule ageing [2]. In recent years, much attention has been focused on solidification of these liquid systems, with particular emphasis on introducing inert solid pharmaceutical excipients [3–7].

Various types of solid carriers have been extensively investigated to solidify SMEDDS, including silica-based water-insoluble adsorbents (e.g., porous silica, magnesium aluminometasilicate, and calcium silicate), cellulose-based hydrophilic diluents (e.g., microcrystalline cellulose, hydroxypropyl cellulose, and low-substituted hydroxypropyl cellulose), and saccharide-based water-soluble diluents (e.g., maltodextrin, lactose, and starch) [7–11]. Although adsorbents with high oil-absorbing capacity minimize the quantity required to solidify the SMEDDS, incomplete desorption of SMEDDS components may occur due to hydrophobic interactions between the drug and adsorbents [8]. Diluents generally possess lower oil-absorbing capacities than adsorbents, but could allow complete desorption of SMEDDS components [7, 9]. Therefore, a suitable combination of adsorbent and diluent is desirable for developing a solidified SMEDDS, while minimizing the total mass and enhancing drug dissolution. However, until now, no attempts have been made to simultaneously use both adsorbents and diluents for the solidification of SMEDDS.

For developing tablet dosage forms, choice of components and compaction procedure is important. In particular, the role of disintegrating agents is crucial in the formulation of solidified SMEDDS preparations that can rapidly disintegrate and spontaneously emulsify in gastric or intestinal fluid [4, 6]. Solidified tablets containing croscarmellose sodium or Kollidon^®^ CL-SF as a disintegrant showed maximum drug release of over 90% within 30 min, whereas tablets without disintegrant resulted in poor drug release of less than 5% after 2 h [2, 6]. Nano- or micro-particles of solid carrier can also induce greater compaction, thus retarding disintegration [12]. Superdisintegrants such as croscarmellose sodium (CS), sodium starch glycolate (SSG), and Kollidon^®^ CL (KC) have been used in tablet formulation, resulting in good disintegration characteristics [2, 6, 13]. Compression force can also affect the performance of disintegrants. Increased compression force results in increased tablet hardness, thus delaying disintegration [14–16].

The present study was performed to develop a solidified dosage form of VST-containing SuSMEDDS (S-SuSMEDDS), to take advantage of improved drug dissolution and oral absorption properties afforded by solidification of the liquid formulation. First, solid carriers (either adsorbents or diluents) were screened based on solidifying behavior and desorption characteristics. Solidification was then optimized, combining the two solid carriers to obtain S-SuSMEDDS granules by using D-optimal mixture design. Second, S-SuSMEDDS tablets were prepared by direct compression of a mixture of S-SuSMEDDS granules and various superdisintegrants. The tableting procedure was optimized using 3-level factorial design (3-LFD), involving compression force, and the concentration and type of superdisintegrant. Finally, in addition to a dissolution comparison, *in vivo* pharmacokinetic (PK) assessments were carried out in rats.

## RESULTS AND DISCUSSION

Previously, we demonstrated that a supersaturable SMEDDS (SuSMEDDS) formulation greatly improved the dissolution and oral bioavailability of VST, while reducing the total volume required for treatment [1]. In the present study, the development of a solidified SuSMEDDS (S-SuSMEDDS) will be discussed in terms of solid carrier screening, optimization of solid formulation, *in vitro* characterization including dissolution, and *in vivo* PK evaluation.

### Solid carrier selection

To develop a solidified formulation of SuSMEDDS, selection of a suitable solid carrier is a key for effective adsorption and absorption of the liquid components. Different types of solid carriers have varied characteristics, such as differences in particle size, pore size, specific surface area, and oil-absorption capacity, thereby affecting the solidification of SuSMEDDS. In this experiment, various types of solid carrier were screened: Sylysia^®^ 350, Neusilin^®^ US2, and Florite^®^ PS-10 as silica-based adsorbents, owing to their small particle size, high surface area, uniform porous structure, and high oil-absorption capacity; Vivapur^®^ 105, hydroxypropyl cellulose L type (HPC), and low-substituted hydroxypropyl cellulose B1 (L-HPC) as cellulose-based diluents, owing to their relatively hydrophilic and viscous properties; and Starch^®^ 1500, lactose monohydrate, and maltodextrin as saccharide-based diluents, as they are water-soluble and often used in commercial products.

**Figure 1:**
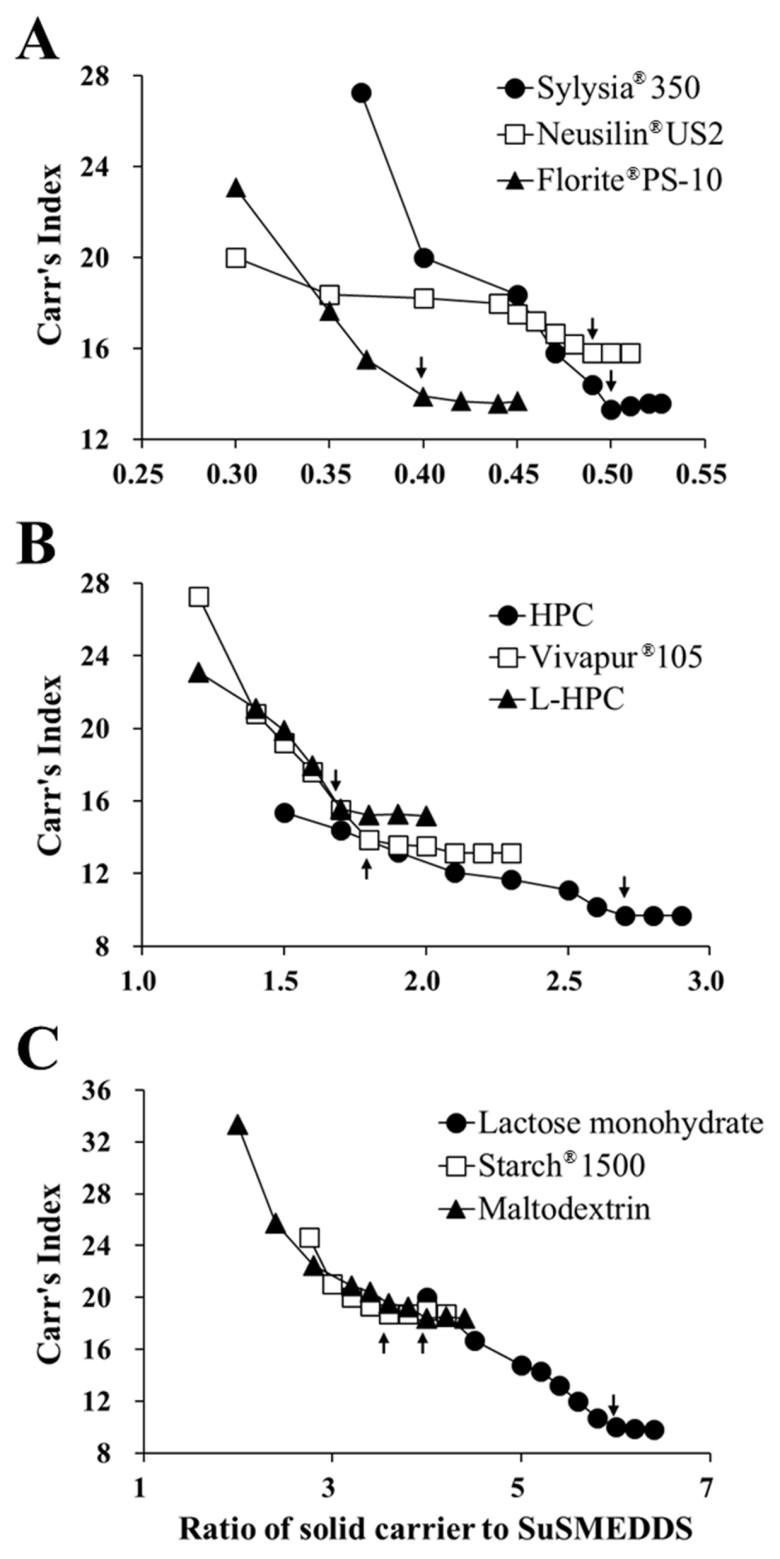
Flow property changes in the S-SuSMEDDS formulation against the ratio of solid carriers to SuSMEDDS Arrow indicates the critical point representing a constant flow property (CSR). **(A)** Silica-based adsorbents. **(B)** Cellulose-based diluents. **(C)** Saccharide-based diluents.

Figure 1 represents the flow properties of the solidified mass in terms of Carr’s index (CI) against the ratio of solid carrier to SuSMEDDS. Flow properties of all of the S-SuSMEDDS increased as the ratio of solid carrier to SuSMEDDS increased. USP guidelines classify the flow property in terms of CI values, as excellent (1–10), good (11–15), fair (16–20), passable (21–25), and poor (>26) [17]. CI values for all of the S-SuSMEDDS were observed within the range of 9.2–18.8%, indicating that SuSMEDDS were suitably absorbed into the solid carriers, and thus the resultant S-SuSMEDDS showed fair to excellent free-flowing properties. Solidifying behavior was further evaluated by critical solidifying ratio (CSR), a critical value representing constant free-flow, as listed in Table 1. The following values were observed, in order: silica-based adsorbents (0.4–0.5) > cellulose-based diluents (1.7–2.7) > saccharide-based diluents (3.6–6.0), demonstrating superior SuSMEDDS-absorption capacities of adsorbents compared to diluents. Saccharide-based diluents showed inferior solidifying properties compared with cellulose-based diluents and silica-based adsorbents, in which hydrophobic interactions and/or van der Waals forces between solid carriers and SuSMEDDS may play an important role for solidification [18]. Moreover, CSR values for silica-based adsorbents were much lower than those of cellulose-based diluents. Since silica-based adsorbents have a mesoporous structure and intra-particle air spaces (void volume), they potentially provide a greater surface area for SuSMEDDS contact compared with cellulose-based diluents, thus absorbing a higher quantity of SuSMEDDS. Among the adsorbents, Florite^®^ PS-10 showed the lowest CSR value, and could therefore be a promising candidate for solid carrier selection.

**Table 1:** Solid carrier screening for the solidification of SuSMEDDS

	Silica-based adsorbents			Cellulose-based diluents			Saccharide-based diluents		
	Sylysia^®^ 350	Neusilin^®^ US2	Florite^®^ PS-10	HPC	Vivapur^®^ 105	L-HPC	Lactose	Starch^®^ 1500	Maltodextrin
*Composition* (mg)									
SuSMEDDS^a^	224.8	224.8	224.8	224.8	224.8	224.8	224.8	224.8	224.8
Solid carrier	112.3	110.1	89.9	606.6	404.4	381.9	1347.9	808.7	898.6
Total	337.1	334.9	314.7	831.4	629.2	606.7	1572.7	1033.5	1123.4
*Physical characteristics*^b^									
Droplet size (nm)	147.7 ± 9.1	120.0 ± 7.1	146.4 ± 1.1	130.5 ± 4.4	151.9 ± 9.4	168.1 ± 8.2	151.5 ± 3.6	145.6 ± 2.2	121.2 ± 56.6
PDI	0.36 ± 0.05	0.41 ± 0.05	0.42 ± 0.11	0.57 ± 0.09	0.21 ± 0.03	0.47 ± 0.05	0.52 ± 0.55	0.49 ± 0.06	0.74 ± 0.07
Drug content (%)	93.7 ± 1.0	94.5 ± 2.5	94.1 ± 3.1	101.4 ± 6.6	98.6 ± 4.0	102.4 ± 2.6	99.6 ± 5.6	104.9 ± 3.4	98.2 ± 2.9
CSR (g/mL)	0.5	0.49	0.4	2.7	1.8	1.7	6	3.6	4
DE (%)^c^	40.6 ± 2.5	46.3 ± 1.7	46.5 ± 2.5	56.3 ± 1.6	66.3 ± 2.1	55.4 ± 0.5	48.1 ± 1.1	48.9 ± 2.1	50.4 ± 2.8

^a^SuSMEDDS composed of VST (80 mg), Capmul^®^ MCM (13.2 mg), Tween^®^ 80 (59.2 mg), Transcutol^®^ P (59.2 mg), and Poloxamer 407 (13.2 mg) was formulated based on the earlier report [1]. ^b^Values are presented as the mean ± SD (n = 3). ^c^Dissolution efficiency (DE) was calculated using the trapezoidal rule as follows: $\text{DE}\left(\%\right)=\left[\int_{\text{t}1}^{\text{t}2}\text{y dt}/{\text{y}}_{100}\left(\text{t}2-\text{t}1\right)\right]\times 100$, where y is the percentage of dissolved product [42].

Abbreviations: SuSMEDDS, supersaturable self-microemulsifying drug delivery system; HPC, hydroxypropyl cellulose; L-HPC, low-substituted hydroxypropyl cellulose; PDI, polydispersity index; CSR, critical solidifying ratio.

Drug content and droplet size upon reconstitution of the VST-loaded solidified mass are listed in Table 1. The drug content of all preparations was in the range of 93–105%, suggesting that the VST-containing SuSMEDDS was efficiently absorbed onto the solid carriers. Droplet size was observed to be in the range of 120.0–168.1 nm for all preparations. These sizes are very close to those observed for SuSMEDDS (187.8 ± 5.5 nm), as reported previously [1]. It was therefore expected that the components of the SuSMEDDS such as oil, surfactant, co-surfactant, and supersaturating agent could be amply desorbed from solid carriers into the medium, thus maintaining the characteristics of the SuSMEDDS. Solid carrier type did not appear to impact reconstitution properties.

The dissolution efficiency (DE) values of various solidified masses were evaluated in pH 1.2 medium to support solid carrier selection, and the results suggested a dependence on solid carrier type. DE values were as follows, in order: cellulose-based diluents (55.4–66.3%) > saccharide-based diluents (48.1–50.4%) > silica-based adsorbents (40.6–46.5%). In comparison to DE values measured for SuSMEDDS (53.6 ± 3.1%), values observed for silica-based adsorbents were considerably lower. This may have been due to the formation of hydrogen bonds between the hydroxyl group of VST and the silanol moiety on the surface of silica-based adsorbents. In contrast, cellulose-based diluents showed slightly higher DE values than SuSMEDDS. The porous structure of cellulose-based diluents may have caused entrapment of SuSMEDDS within the pores, thereby stabilizing the S-SuSMEDDS formulation by inhibiting recrystallization of the drug [19, 20]. In addition, the cellulose-based diluents may have gradually desorbed the components of SuSMEDDS into the medium, thereby enhancing the fine dispersion of SuSMEDDS droplets. The most effective cellulose-based solid carrier for increasing drug release was Vivapur^®^ 105, followed by L-HPC and HPC. This corresponds with particle size: Vivapur^®^ 105 (10 μm) < L-HPC (50 μm) < HPC (<850 μm). As particle size decreases, the solid carrier disperses more evenly, increasing the surface area in the dissolution medium [21]. Florite^®^ PS-10 and Vivapur^®^ 105 showed the highest DE values for the adsorbents and diluents, respectively. It is noteworthy that the adsorbents render high oil-absorbing capacity with incomplete desorption, but the diluents render low oil-absorbing capacity with complete desorption [7, 8]. Thus, in consideration of mutual compensation, a mixed solid carrier, composed of both Florite^®^ PS-10 and Vivapur^®^ 105, was selected for further optimization of solidification.

### Optimization and characterization of S-SuSMEDDS granule

D-optimal mixture design was applied to determine the optimal mixture of the two solid carriers, for minimizing the total mass of the final S-SuSMEDDS granules, while maximizing the oral absorption of VST. Florite^®^ PS-10 (X_1_; mg) and Vivapur^®^ 105 (X_2_; mg) were the independent variables, as listed in Table 2. The CSR (Y_1_; g/mL) and percentage of drug released in 15 min (D_15_, Y_2_;%) were introduced as response variables, due to their crucial roles in determining the absorption and desorption capacity of the solid carrier from the S-SuSMEDDS formulation. As shown in Table 3, for the eight experimental runs, Y_1_ and Y_2_ were in the ranges of 0.42–1.8 g/mL and 45.7–64.3%, respectively. Statistical parameters analyzed using Design-Expert software are listed in Table 4. As all responses were simultaneously fitted to linear, quadratic, and cubic models, the linear model was selected as the best fitting mathematical model for both Y_1_ and Y_2._ Sequential p-values for Y_1_ and Y_2_ were < 0.01, indicating that the effects of the responses were statistically significant up to a 99% confidence level. The lack of fit p-values of the responses Y_1_ and Y_2_ were > 0.1, suggesting an adequate model fit [22]. All squared correlation coefficient (R^2^) and adjusted R^2^ values for Y_1_ and Y_2_ were > 95%, indicating satisfactory analysis quality. Based upon the results obtained from the multiple linear regression analysis for each response variable, the model equations were generated as follows:

Y_1_ = 0.0042 X_1_ + 0.018 X_2_

Y_2_ = 0.467 X_1_ + 0.636 X_2_

**Table 2:** Variables used for the optimization of S-SuSMEDDS granules and tablets, using D-optimal mixture design and 3-LFD, respectively

Type	Variables				Responses	
	Formulation variables	Level used			Response variables	Target
S-SuSMEDDS granules	X_1_ = Florite^®^ PS-10(%)	0 100			Y_1_ = CSR (g/mL)	Minimize
	X_2_ = Vivapur^®^ 105 (%)	0 100			Y_2_ = D_15_ (%)	Maximize
S-SuSMEDDS tablets	X_1_ = Compression force (kgf)	500	1250	2000	Y_1_ = Hardness (N)	> 50
	X_2_ = Concentration of superdisintegrant (%)	2	5	8	Y_2_ = Disint%(%)	Maximize
	X_3_ = Type of superdisintegrant	CS	KC	SSG	Y_3_ = DE (%)	Maximize
					Y_4_ = Total mass (mg)	Minimize

Abbreviations: S-SuSMEDDS, solidified supersaturable self-microemulsifying drug delivery system; 3-LFD, 3-level factorial design; CSR, critical solidifying ratio; D_15_, percentage of drug released in 15 min; Disint%, percentage of disintegration in 30 min; DE, dissolution efficiency; CS, croscarmellose sodium; KC, Kollidon^®^ CL; SSG, Sodium starch glycolate.

**Table 3:** Composition and observed responses from runs

Mixture number	Formulation composition			Response measured			
	X_1_	X_2_	X_3_	Y_1_	Y_2_	Y_3_	Y_4_
*(A) Optimized S-SuSMEDDS granules*							
	Florite^®^ PS-10 (%)	Vivapur^®^ 105 (%)	N.A.^a^	CSR (g/mL)	D_15_ (%)	N.A.	N.A.
1	0	100	-	1.8	64.3	-	-
2	25	75	-	1.45	59.3	-	-
3	50	50	-	1.1	55.5	-	-
4	75	25	-	0.8	52.7	-	-
5	100	0	-	1.4	46.6	-	-
6	0	100	-	1.8	62.8	-	-
7	50	50	-	1.15	54.4	-	-
8	100	0	-	0.42	45.7	-	-
*(B) Optimized S-SuSMEDDS tablets*							
	Compression force (%)	Conc. of super-disintegrant (%)	Type of super-disintegrant	Hardness (N)	Disint (%)	DE (%)	Total mass (mg)
1	500	2	CS	77	23.8	3.4	466.1
2	1250	2	CS	93	23.0	3.3	465.5
3	2000	2	CS	66	23.0	3.1	466.2
4	500	5	CS	76	89.2	27.3	478.2
5	1250	5	CS	72	84.9	28.8	480.5
6	2000	5	CS	73	87.2	28.8	476.6
7	500	8	CS	75	100.0	38.6	489.6
8	1250	8	CS	77	100.0	39.5	489.7
9	2000	8	CS	71	100.0	38.9	493.1
10	500	2	KC	74	16.9	3.8	464.3
11	1250	2	KC	81	18.3	4.3	461.8
12	2000	2	KC	69	19.1	5.3	466.5
13	500	5	KC	80	63.8	32.2	476.3
14	1250	5	KC	88	59.8	33.8	479.3
15	2000	5	KC	82	62.6	31.7	480.5
16	500	8	KC	74	89.5	42.8	493.8
17	1250	8	KC	84	90.1	41.3	490.2
18	2000	8	KC	80	92.0	40.6	489.9
19	500	2	SSG	68	23.8	4.5	461.3
20	1250	2	SSG	86	27.3	4.8	463.7
21	2000	2	SSG	69	26.4	4.8	465.8
22	500	5	SSG	67	41.1	12.1	476.1
23	1250	5	SSG	79	44.1	12.3	479.3
24	2000	5	SSG	66	49.0	11.7	480.2
25	500	8	SSG	76	68.6	25.7	489.3
26	1250	8	SSG	88	69.2	25.6	491.7
27	2000	8	SSG	70	71.1	27.4	493.2
28	1250	5	CS	76	80.1	30.1	478.6
29	1250	5	KC	82	64.4	35.6	479.8
30	1250	5	SSG	83	48.8	26.1	476.5

^a^Not applicable

Abbreviations: S-SuSMEDDS, solidified supersaturable self-microemulsifying drug delivery system; CSR, critical solidifying ratio; D_15_, percentage of drug released in 15 min; Disint%, percentage of disintegration in 30 min; DE, dissolution efficiency; CS, croscarmellose sodium; KC, Kollidon^®^ CL; SSG, Sodium starch glycolate.

**Table 4:** Summary of the results of statistical analyses and model equations for the measured responses

Models	Sequential p-value	Lack of fit p-value	SD	R^2^	Adjusted R^2^	Remark
*(A) Optimized S-SuSMEDDS granules*						
CSR (Y_1_; g/mL)						
Linear	<0.0001	0.4495	0.023	0.9985	0.9983	Suggested
Quadratic	0.2599	0.4751	0.022	0.9989	0.9984	-
Cubic	0.1940	0.8154	0.019	0.9993	0.9988	-
D_15_ (Y_2_; %)						
Linear	<0.0001	0.3206	1.00	0.9818	0.9788	Suggested
Quadratic	0.5592	0.2389	1.05	0.9831	0.9764	-
Cubic	0.2133	0.2487	0.95	0.9809	0.9458	-
*(B) Optimized S-SuSMEDDS tablets*						
Hardness (Y_1_; N)						
Linear	0.6210	0.1014	7.24	0.0964	-0.0482	-
2FI	0.8409	0.0825	7.72	0.1791	-0.1903	-
Quadratic	0.0008	0.2022	5.46	0.6306	0.4049	Suggested
Cubic	0.1307	0.2952	4.45	0.8634	0.6037	Aliased
Disint%(Y_2_; %)						
Linear	<0.0001	0.0377	10.19	0.8926	0.8754	-
2FI	0.0703	0.0519	8.98	0.9333	0.9032	-
Quadratic	0.0046	0.1008	7.02	0.9633	0.9409	Suggested
Cubic	0.0001	0.8308	2.59	0.9972	0.9919	Aliased
DE (Y_3_;%)						
Linear	<0.0001	0.0459	5.15	0.8893	0.8716	-
2FI	0.1163	0.0577	4.69	0.9267	0.8937	-
Quadratic	0.0046	0.1113	3.66	0.9597	0.9350	Suggested
Cubic	0.0003	0.7769	1.48	0.9964	0.9895	Aliased
Total mass (Y_4_; mg)						
Linear	<0.0001	0.4017	1.71	0.9777	0.9675	Suggested
2FI	0.2150	0.4411	1.62	0.9840	0.9596	-
Quadratic	0.5866	0.4168	1.66	0.9849	0.9518	-
Cubic	0.7812	0.2969	1.84	0.9896	0.8594	Aliased

Abbreviations:SD, standard deviation; R^2^, squared correlation coefficient; S-SuSMEDDS, solidified supersaturable self-microemulsifying drug delivery system; CSR, critical solidifying ratio; D_15_, percentage of drug released in 15 min; Disint%, percentage disintegration in 30 min; DE, dissolution efficiency.

**Figure 2:**
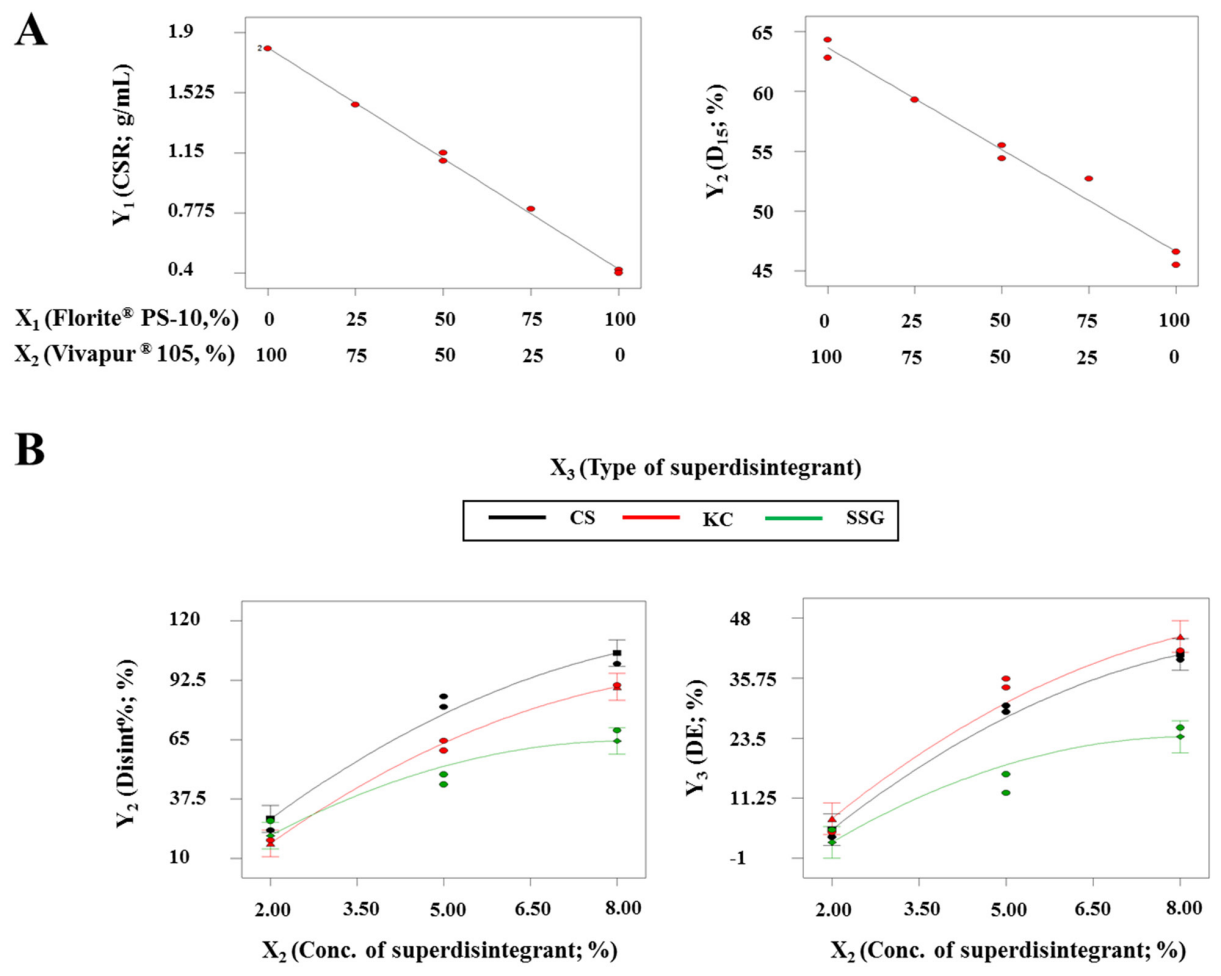
Effect of variables on responses for developing optimized S-SuSMEDDS granules and tablets **(A)** For optimizing the S-SuSMEDDS granules: two-component mixture plots for the effect of variables (X_1_ and X_2_) on responses Y_1_ and Y_2_. **(B)** For optimizing the S-SuSMEDDS tablets: interaction plots for the effect of variables (X_2_ and X_3_) on responses Y_2_ and Y_3_ with a fixed X_1_ value (1250 kgf).

The interaction term (X_1_X_2_) was not observed in the above model equations, suggesting that there was no significant interaction effect between X_1_ and X_2_ on Y_1_ and Y_2_. The similar particle sizes of X_1_ and X_2_ (Florite^®^ PS-10, 10 μm; Vivapur^®^ 105, 15 μm) may have formed a homogeneous mixture of X_1_ and X_2_, permitting equal absorption of SuMEDDS into either X_1_ or X_2_. Figure 2A shows the effects of X_1_ and X_2_ on the responses. As X_1_ increased and X_2_ decreased, both the CSR value (Y_1_) and D_15_ (Y_2_) constantly decreased in the ranges of 1.8–0.4 g/mL and 64.3–45.7%, respectively. As expected, X_1_ may reduce the quantity required for solidifying the SuSMEDDS, but adversely affects the dissolution rate of VST, compared with X_2_. These data indicated that each solid carrier maintained physiochemical properties to absorb and desorb the components of SuSMEDDS, even though the SuSMEDDS was homogenously absorbed into the mixtures of X_1_ and X_2_.

The independent variables were simultaneously optimized for responses using the desirability function. As shown in Table 2, Y_1_ was set to be minimized, whereas Y_2_ was set to be maximized. The optimized amounts of Florite^®^ PS-10 (X_1_) and Vivapur^®^ 105 (X_2_) were 53% and 47%, respectively, with a corresponding desirability value of 0.502. Predicted and experimental values were compared to determine the accuracy of prediction, using percentage prediction error (Table 5). The calculated percentage prediction errors were low (<10%), indicating that the D-optimal mixture design used to optimize the composition of mixed solid carriers was accurate and reliable.

**Table 5:** Experimental and predicted values for the optimized S-SuSMEDDS granules and tablets

Type	Composition	Response	Importance	Predicted value	Experimental value	Percentage prediction error^a^
Optimized S-SuSMEDDS granules	X_1_: 53%	Y_1_ (mL/100 g)	+	1.07	1.0 ± 0.2	-7.0
	X_2_: 47%	Y_2_ (%)	+	54.6	58.6 ± 2.2	6.8
Optimized S-SuSMEDDS tablets	X_1_: 536 kgf	Y_1_ (N)	-	73.4	76.6 ± 3.5	4.2
	X_2_: 6.9%	Y_2_ (%)	++	98	94.3 ± 5.6	-3.8
	X_3_: CS	Y_3_ (%)	+++	36.1	38.5 ± 1.8	6.2
		Y_4_ (mg)	+	485.8	489.4 ± 2.6	0.7

^a^Calculated using the formula ([experimental value - predicted value]/experimental value) × 100 (%). Values are presented as the mean ± SD (n = 3).

Abbreviations: S-SuSMEDDS, solidified supersaturable self-microemulsifying drug delivery system; CS, croscarmellose sodium.

**Figure 3:**
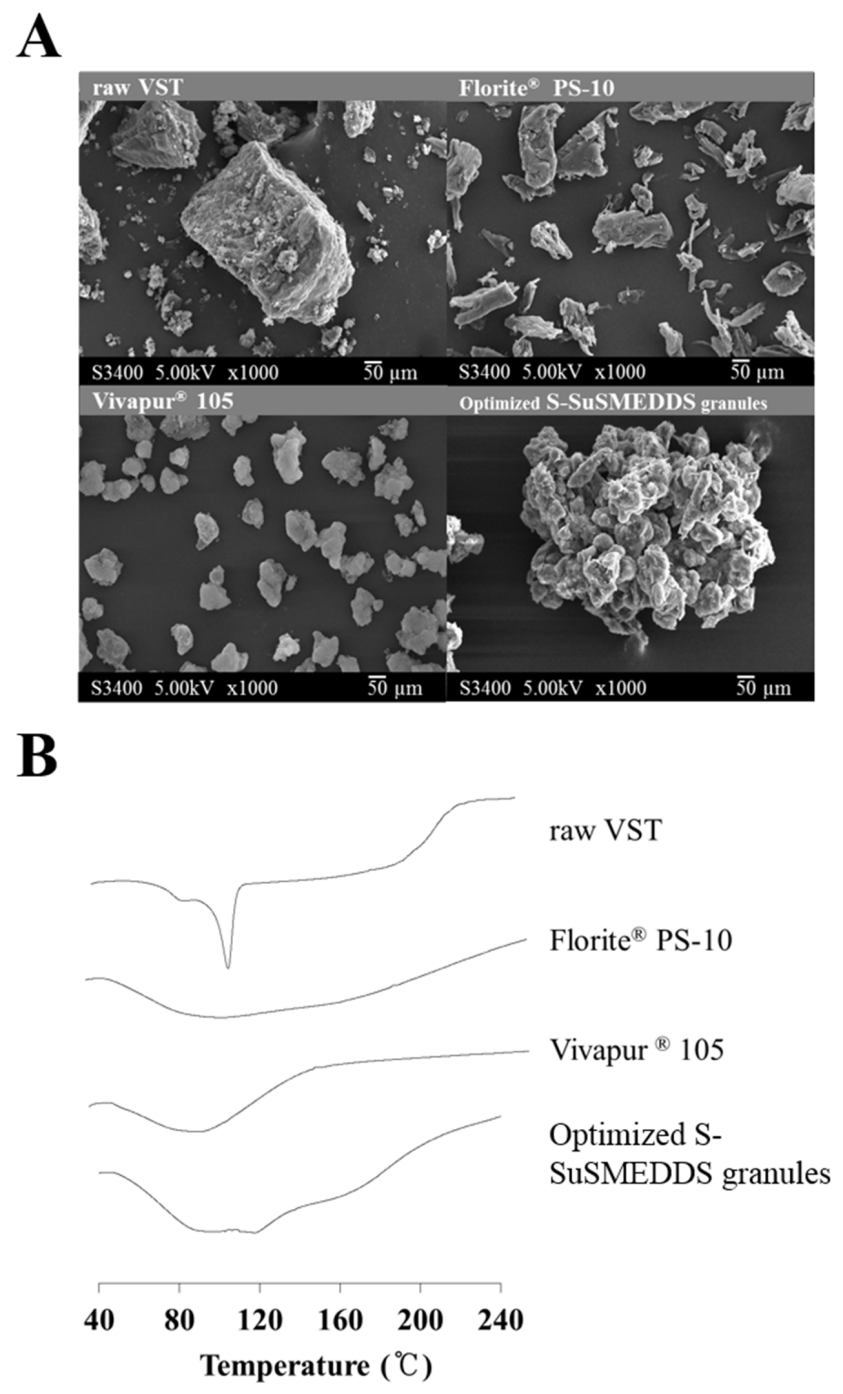
Solid-state properties of raw VST, solid carriers (Florite^®^ PS-10 and Vivapur^®^ 105), and VST-loaded optimized S-SuSMEDDS granules **(A)** Scanning electron microscopic images. **(B)** Differential scanning calorimetric thermograms.

As shown in Figure 3A, scanning electron microscopy (SEM) images exhibited the morphologies of VST, two solid carriers, and optimized S-SuSMEDDS granules. VST as a raw material appeared to have a rough and rectangular-shaped crystalline structure [23]. Solid carriers were identified as aggregates of particles. No rectangular crystals of VST were observed on the surface of the optimized S-SuSMEDDS granule, indicating that VST-loaded SuSMEDDS was completely absorbed onto the solid carriers. To identify the crystalline state of VST in the solidified formulation, differential scanning calorimetry (DSC) was performed. DSC thermograms of VST, the two solid carriers, and optimized S-SuSMEDDS granules are shown in Figure 3B. VST has a sharp endothermic peak at about 110°C, corresponding to its melting point and indicating its crystalline nature. There were no specific peaks for the solid carriers Florite^®^ PS-10 and Vivapur^®^ 105. Optimized S-SuSMEDDS granules also lacked a typical VST peak, suggesting that VST exists in a solubilized and/or amorphous form in SuSMEDDS components.

### Development of S-SuSMEDDS tablets

In a preliminary study, optimized S-SuSMEDDS granules were easily converted to tablet form, but this resulted in poor dissolution of less than 5% drug release after 2 h, with no disintegration. This may be attributable to the high hardness of the tablet induced by the inclusion of Vivapur^®^ 105, which is often regarded as one of the best binders for direct compression [5, 24]. Our previous effort to convert optimized S-SuSMEDDS granules to tablet form, without the use of Vivapur^®^ 105, was unfortunately unsuccessful due to the low hardness, the sticking phenomenon, or a combination of both.

To reduce disintegration time and improve the dissolution of VST, superdisintegrants can be used in tablet development. In comparison to conventional disintegrants, superdisintegrants such as CS, KC, and SSG show good disintegration action at low concentrations, ensuring fragmentation of the tablet dosage form upon ingestion and allowing the onset of drug dissolution and eventual absorption [25]. It is important to determine the most suitable superdisintegrants for the S-SuSMEDDS tablet preparation. The main mechanisms for disintegration differ: swelling for SSG [26]; swelling, wicking, and strain recovery for CS [27]; and wicking followed by secondary swelling for KC [28, 29].

However, regardless of superdisintegrant type, compression force may affect disintegration performance of the S-SuSMEDDS formulation. Several studies reported that high compression force increased tablet hardness, resulting in an increase in disintegration time [14–16]. This may be due to the effect of compression force on tablet porosity. High compression force decreases intermolecular voids and increases inter-particle bonding and tablet densification, potentially inducing increased tablet hardness and tensile strength [30]. Thus, when considering these factors, the inclusion of compression force, and superdisintegrant concentration and type, in the optimization process was necessary for successful development of tablet dosage forms.

### Experimental design of S-SuSMEDDS tablets

3-LFD was applied to determine the optimal composition of superdisintegrant and compression force, to promote disintegration and drug dissolution in the optimized S-SuSMEDDS tablets. Compression force (X_1_; kgf) and superdisintegrant concentration (X_2_;wt% of S-SuSMEDDS granule) were chosen as numeric independent variables, while superdisintegrant type (X_3_) was selected as a categoric independent variable, as listed in Table 2. Hardness (Y_1_; N) was introduced as a response variable to evaluate suitability of the formulation for tablet dosage form. The percentage of disintegration in 30 min (Disint%, Y_2_; %) and DE (Y_3_;%) were established as response variables to determine disintegration capacity, in order to improve drug dissolution. Total mass (Y_4_; mg) was chosen as an additional response variable to satisfy the goal of minimizing S-SuSMEDDS tablet mass. As shown in Table 3, for the 30 experimental runs, Y_1_, Y_2_, and Y_3_ were observed to be in the ranges of 66–93 N, 16.9–100.0%, and 461.3–493.8 mg, respectively. Statistical parameters analyzed using Design Expert software are listed in Table 4. The quadratic model was suggested as the best fitting mathematical model for Y_1_, Y_2_, and Y_3_ by comparing several statistical parameters. Y_4_ was selected for the linear model. The responses of Y_2_, Y_3_, and Y_4_ showed reasonably suitable statistical parameters, including p-values < 0.05, lack of fit p-values > 0.1, and R^2^ > 0.95, indicating significant model terms and accurate, reliable model fit. However, R^2^ value of 0.6306 was obtained for Y_1_ using the suggested model, indicating unsatisfactory analysis. Therefore, the model fit of Y_1_ may potentially have little impact on the optimization process. Results of the multiple linear regression analyses for each response variable were derived using the best fitting models as follows:

Y_1_ (CS) = 68.24 + 0.04 X_1_ – 2.53 X_2_ + 0.0004 X_1_X_2_ – 0.00002 X_1_^2^ + 0.13 X_2_^2^

Y_1_ (KC) = 58.71 + 0.04 X_1_ – 1.03 X_2_ + 0.0004 X_1_X_2_ – 0.00002 X_1_^2^ + 0.13 X_2_^2^

Y_1_ (SSG) = 57.85 + 0.04 X_1_ – 1.19 X_2_ + 0.0004 X_1_X_2_ – 0.00002 X_1_^2^ + 0.13 X_2_^2^

Y_2_ (CS) = -19.22 + 0.004 X_1_ + 11.62 X_2_ – 0.00006 X_1_X_2_ – 0.000002 X_1_^2^ – 0.56 X_2_^2^

Y_2_ (KC) = -16.65 + 0.004 X_1_ + 11.85 X_2_ – 0.00006 X_1_X_2_ – 0.000002 X_1_^2^ – 0.56 X_2_^2^

Y_2_ (SSG) = -17.10 + 0.004 X_1_ + 9.25 X_2_ – 0.00006 X_1_X_2_ – 0.000002 X_1_^2^ – 0.56 X_2_^2^

Y_3_ (CS) = -19.23 + 0.004 X_1_ + 11.62 X_2_ – 0.00006 X_1_X_2_ – 0.000002 X_1_^2^ – 0.56 X_2_^2^

Y_3_ (KC) = -16.65 + 0.004 X_1_ + 11.85 X_2_ – 0.00006 X_1_X_2_ – 0.000002 X_1_^2^ – 0.56 X_2_^2^

Y_3_ (SSG) = -17.1 + 0.004 X_1_ + 9.25 X_2_ – 0.00006 X_1_X_2_ – 0.000002 X_1_^2^ – 0.56 X_2_^2^

Y_4_ (CS/KC/SSG) = 454 + 0.001 X_1_ + 4.44 X_2_

Results of analysis of variance for the responses Y_2_ and Y_3_ are listed in Table 6. P-values of > 0.05 for X_1_, X_1_X_2_, X_1_X_3_, and X_1_^2^ indicated that X_1_ had an insignificant effect on Y_2_ and Y_3_. In the S-SuSMEDDS formulation, compression force was shown to have little influence on disintegration performance and drug dissolution. The porosity of solid carriers may be mostly negated due to absorption of the liquid formulation, leading to the diminished effect of compression force. However, p-values of < 0.05 for X_2_, X_3_, X_2_X_3_, and X_2_^2^ indicated that X_2_ and X_3_ had significant effects on Y_2_ and Y_3_. Figure 2B represents the effects of varying superdisintegrant concentration (X_2_) and type (X_3_) with a fixed value of X_1_. Disint% (Y_2_) increased from 16.8% to 100.0% as X_2_ increased and X_3_ changed. The increment of Disint% was in the order of CS > KC > SSG. This suggested that the wicking mechanism utilized by CS and KC was more favorable for disintegration of the S-SuSMEDDS tablets than the swelling mechanism proposed for SSG. Swelling is related to dimensional amplification, where particles extend omni-directionally to push apart the adjoining components and progress the degradation of the tablet matrix, while wicking is defined as a process of liquid entry by capillarity into micro-structured crevices within the tablet matrix [31, 32]. Tablet porosity contributes considerably to the performance of swelling disintegrants [25]. The porous structure of the S-SuSMEDDS filled with liquid formulation may hinder liquid entry and prolong disintegration time. However, wicking may induce water imbibition within the S-SuSMEDDS tablet, resulting in rapid desorption of the components and an increase in disintegration performance. S-SuSMEDDS tablets with added CS showed better disintegration performance than S-SuSMEDDS tablets with added KC. Wicking and swelling are recognized as the probable disintegration mechanisms for both CS and KC, while strain recovery is proposed to be utilized only by CS [27–29]. This indicates that strain recovery may enhance the disintegration performance of S-SuSMEDDS tablets. Strain recovery is a reversible viscoelastic process of deformation: upon contact of compacted tablets with aqueous media, this process allows mechanical activation of disintegrant polymer chains which may assist in partial recovery of their original shapes [33]. In addition, DE (Y_3_) increased from 3.1% to 42.8% as X_2_ increased and X_3_ altered. Consistent with Disint%, both S-SuSMEDDS with added CS and S-SuSMEDDS tablets with added KC showed significantly higher DE values than S-SuSMEDDS tablets with added SSG. However, the difference in DE values between formulations with added KC and CS was insignificant.

**Table 6:** Analysis of variance for quadratic model of the measured responses for developing an optimized S-SuSMEDDS tablet

Source	DF	Y_2_ (Disint%)			Y_3_ (DE)		
		SS	F	p-value	SS	F	p-value
Model	11	2115.85	42.99	<0.0001	5748.42	38.98	<0.0001
X_1_	1	10.73	0.22	0.6461	0.2	0.015	0.904
X_2_	1	18622.53	378.36	<0.0001	4452.53	331.85	<0.0001
X_3_	2	1466.2	29.79	<0.0001	874.11	32.57	<0.0001
X_1_X_2_	1	0.07	0.001	0.9703	0.24	0.018	0.8949
X_1_X_3_	2	10.62	0.22	0.808	0.84	0.031	0.9692
X_2_X_3_	2	480.4	9.76	0.0013	222.91	8.31	0.0028
X_1_^2^	1	3.17	0.064	0.8025	5.17	0.39	0.5425
X_2_^2^	1	719.15	14.61	0.0012	176.84	13.18	0.0019
Residual	18	885.95			241.51		
Lack of fit	15	852.95	5.17	0.1008	231.83	4.79	0.1113
Pure error	3	33			99.69		
Corrected total	29	24160.32			5989.93		

Abbreviations:DF, degrees of freedom; SS, sum of squares; S-SuSMEDDS, solidified supersaturable self-microemulsifying drug delivery system; Disint%, percentage disintegration in 30 min; DE, dissolution efficiency.

### Optimization of S-SuSMEDDS tablets using the desirability function

**Figure 4:**
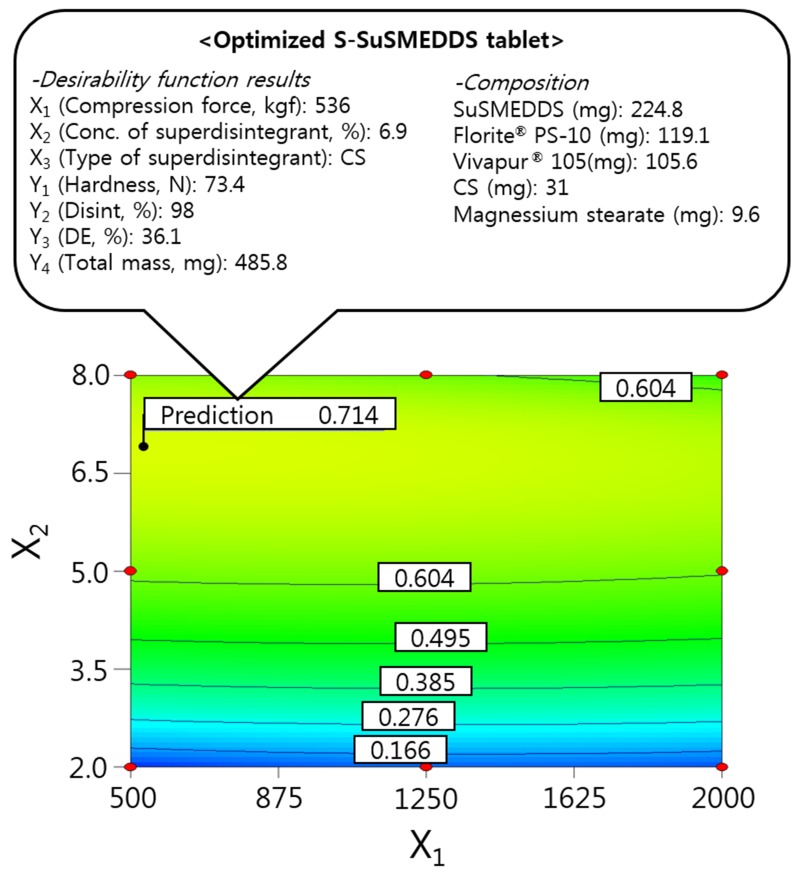
Overlay plot of the optimized S-SuSMEDDS tablet Values in contour lines represent the desirability.

The independent variables were simultaneously optimized for responses using the desirability function. As shown in Table 2, Y_1_ was set at > 50 N for good hardness, Y_2_ and Y_3_ were maximized to improve oral absorption of VST, and Y_4_ was minimized to reduce the mass of individual S-SuSMEDDS tablets. Dependent variables were then ordered by importance (Y_3_ > Y_2_ > Y_4_(Table 5)), to achieve optimal desirability. Optimized compression force (X_1_), superdisintegrant concentration (X_2_), and superdisintegrant type (X_3_) were 536 kgf, 6.9%, and CS, respectively, with a corresponding desirability value of 0.714. The correlating predicted responses were 73.4 N (Y_1_), 98% (Y_2_), 36.1% (Y_3_), and 485.8 mg (Y_4_). When equal importance was assigned to all responses, the corresponding desirability value decreased to 0.617, with predicted responses of 73.3 N (Y_1_), 87% (Y_2_), 30.8% (Y_3_), and 480.9 mg (Y_4_). Optimization with the inclusion of varying levels of importance resulted in significantly higher desirability values, compared with those obtained by performing optimization with responses of equal importance. This suggests that S-SuSMEDDS tablets were successfully optimized, using suitable desirability functions. Consequently, as depicted in Figure 4, the final optimized S-SuSMEDDS tablet was composed of VST-loaded SuSMEDDS (224.8 mg), Florite^®^ PS-10 (119.1 mg), Vivapur^®^ 105 (105.6 mg), croscarmellose sodium (31 mg), and magnesium stearate (9.6 mg). Meanwhile, the percentage prediction errors associated with optimized S-SuSMEDDS tablets were low (<10%), indicating accuracy and reliability of the 3-LFD. Physical properties of the optimized S-SuSMEDDS tablets were evaluated to evaluate preparation success. Drug content was in the range of 94.6–96.1%; tablet diameter and thickness variation was within 5% of the total weight; friability was in the range of 0.07–0.13%, and the weight loss of <1% observed in the friability test was generally acceptable [22]. These results indicate that optimized S-SuSMEDDS tablets were prepared successfully using 3-LFD.

### *In vitro* dissolution profiles

**Figure 5:**
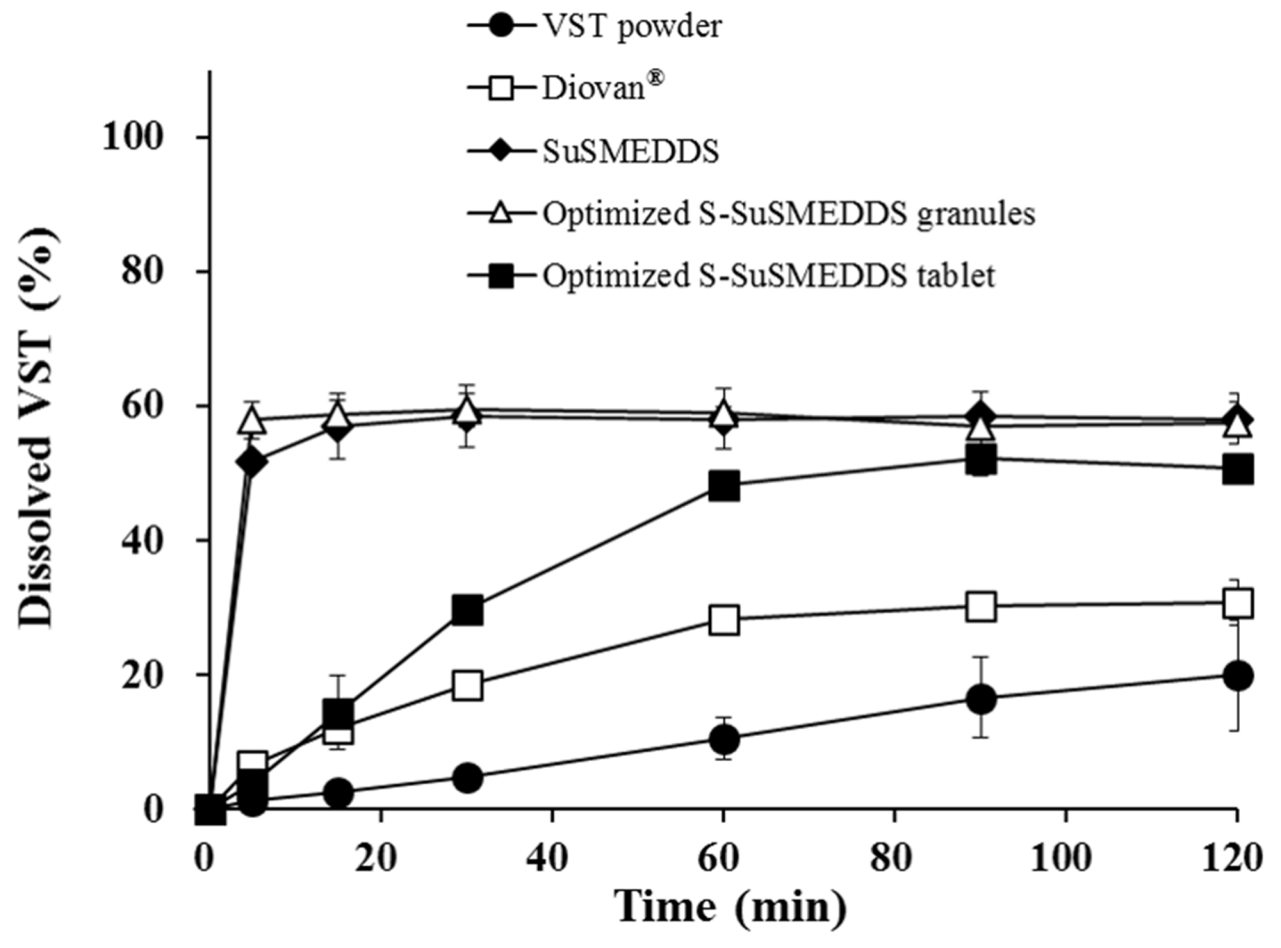
Dissolution profiles of various formulations containing the equivalent of 80 mg VST in pH 1.2 medium Values are presented as the mean ± SD (n = 3).

Dissolution profiles were determined for VST in various formulations, including VST powder, a commercial product (Diovan^®^), optimized S-SuSMEDDS granules, and optimized S-SuSMEDDS tablets, in pH 1.2 medium over a 2 h period (Figure 5). During this period, dissolution of VST powder and Diovan^®^ gradually increased to approximately 20% and 30%, respectively. Fast dissolution was observed for optimized S-SuSMEDDS granules, with up to 60% of the total VST content released in the initial 5 min. These data indicated that components of the SuSMEDDS were rapidly desorbed from Florite^®^ PS-10 and Vivapur^®^ 105, forming microemulsions which enhanced the dissolution of VST. However, optimized S-SuSMEDDS tablets showed an unexpectedly gradual increase in dissolution, resulting in a plateau of >50% dissolution at 60 min, before reaching a similar dissolution level to S-SuSMEDDS granules. Although dissolution of optimized S-SuSMEDDS tablets was slower than that of optimized S-SuSMEDDS granules, this would be unlikely to affect total VST absorption, as the drug is preferentially absorbed in both the stomach and the upper small intestine [34]. Optimized S-SuSMEDDS tablets greatly increased VST dissolution after 2 h by 2.5-fold and 1.6-fold, compared with raw VST powder and Diovan^®^, respectively, suggesting that the S-SuSMEDDS formulation in tablet dosage form would be a promising strategy for improving the solubility of drugs with a low oral bioavailability.

### *In vivo* PK behavior

**Figure 6:**
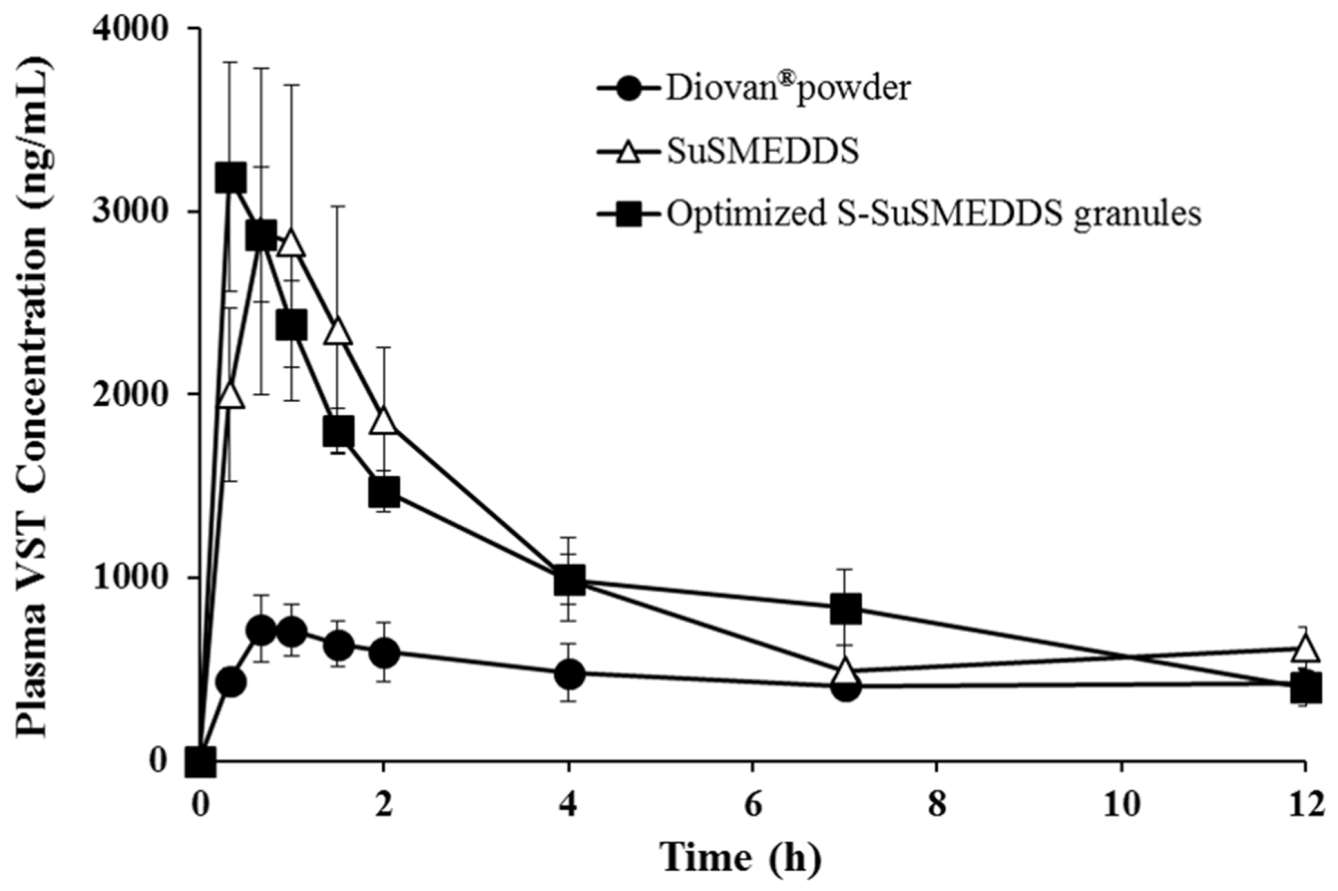
Plasma concentration profiles in rats after oral administrations of various formulations containing an equivalent dose of 10 mg/kg of VST Values are presented as mean ± standard error (n= 6-7).

The PK behavior of VST in rats was evaluated after oral treatment with Diovan^®^ powder, SuSMEDDS, and optimized S-SuSMEDDS granules. Plasma levels of VST were measured and plotted against time (Figure 6). Both SuSMEDDS and optimized S-SuSMEDDS granules showed greater absorption than Diovan^®^ powder. The initial higher absorption for SuSMEDDS and optimized S-SuSMEDDS granules may be attributed to the enhancement of VST dissolution by the acidic conditions [1]. The synergistic effect of oil and surfactants contained in both SuSMEDDS and optimized S-SuSMEDDS granules could improve the oral absorption of VST in the gastric intestinal (GI) tract [35]. The oil-in-water nanoemulsions produced by oil and surfactants could have an active influence on intestinal permeation of both transcellular and paracellular transport and protect the drug from enzyme degradation [36, 37]. In particular, Transutol^®^ P and Tween^®^ 80, contained in both SuSMEDDS and optimized S-SuSMEDDS granules, have been used as permeation-enhancers and/or P-glycoprotein inhibitors, to improve oral absorption of several poorly permeable drugs [35, 38].

PK parameters of the different formulations are listed in Table 7. The maximum plasma concentration (C_max_) and area under the curve (AUC) values of the Diovan^®^ powder were much lower than those of SuSMEDDS and optimized S-SuSMEDDS granules. C_max_ values of SuSMEDDS and optimized S-SuSMEDDS granules were 2.0 and 2.2 times higher, respectively, than that of Diovan^®^ powder. Based on AUC values, the relative bioavailabilities (RBAs) of SuSMEDDS and optimized S-SuSMEDDS granules were 206% and 222%, respectively, compared to that obtained for Diovan^®^ powder. These results indicated that both SuSMEDDS and optimized S-SuSMEDDS granules could efficiently form microemulsions in the GI tract, resulting in the enhanced oral absorption of VST. Meanwhile, the time to reach the maximum plasma concentration (T_max_), C_max_, and AUC values of SuSMEDDS were 1.8, 0.9, and 0.9 times greater, respectively, than those of optimized S-SuSMEDDS granules. In particular, SuSMEDDS in a gelatin capsule yielded a slower and decreased absorption profile than the optimized S-SuSMEDDS formulation, resulting in a T_max_ value of 1.17 h. This value was greater than that found in an earlier study (0.33 h), in which SuSMEDDS was administered in a pre-dispersed concentrated solution [1]. This discrepancy in the rate of absorption may be due to the inability of the highly viscous mass in the gelatin capsule to instantly disperse throughout the medium. In contrast, optimized S-SuSMEDDS granules provide an increased surface area for VST dissolution, subsequently conferring a stable microenvironment for homogenous self-emulsification in the GI tract. Therefore, the optimized S-SuSMEDDS granules described here could be a practical means for developing solid dosage forms of liquefied formulations such SMEDDS and SuSMEDDS, although future PK evaluations of S-SuSMEDDS tablets in animals or human volunteers will be necessary.

**Table 7:** Pharmacokinetic parameters of VST in different formulations in rats

Parameters	Diovan^®^ powder	SuSMEDDS	Optimized S-SuSMEDDS granules
AUC_0-12_ (ng·h/mL)	5561.5 ± 2883.4	11463.3 ± 4523.1^*^	12350.5 ± 3774.5^*^
C_max_ (ng/mL)	849.3 ± 476.5	3120.6 ± 2367.7^*^	3337.9 ± 1396.6^*^
T_max_ (h)	0.93 ± 0.60	1.17 ± 0.66	0.63 ± 0.51
RBA (%)			
*vs.* Diovan^®^	-	206.1	222.1
*vs.* SuSMEDDS	-	-	107.7

^*^Significantly different at *p* < 0.05 versus Diovan^®^ powder. Values are presented as the mean ± SD (n = 6-7).

Abbreviations:VST, valsartan; S-SuSMEDDS, solidified supersaturable self-microemulsifying drug delivery system; AUC, area under the curve; C_max_, peak plasma concentration; T_max_, time to peak plasma; RBA, relative bioavailability; SuSMEDDS, supersaturable self-microemulsifying drug delivery system.

## MATERIALS AND METHODS

### Materials

VST was supplied by Daewon Pharm. Co. Ltd. (Seoul, Korea). Diovan^®^ tablets containing 80 mg VST were purchased as a reference product. Transcutol^®^ P (diethylene glycol monoethyl ether) was supplied by Gattefossé (Saint Priest, France). Capmul^®^ MCM (glyceryl caprylate/caprate) was supplied by Abitec Co. (Janesville, WI, USA). Poloxamer 407 (Pluronic^®^ F-127) and KC were supplied by BASF (Ludwigshafen, Germany). Tween^®^ 80 and maltodextrin were purchased from Sigma-Aldrich (St. Louis, MO, USA). Sylysia^®^ 350 (porous silica) was supplied by Fuji Silysia Chemical Co., Ltd. (Aichi, Japan). Neusilin^®^ US2 (magnesium aluminometasilicate) was supplied by Fuji Chemical Industry Company (Toyama, Japan). Florite^®^ PS-10 (calcium silicate) was supplied by Tomita Pharmaceutical Co., Ltd. (Tokushima, Japan). Vivapur^®^ 105 (microcrystalline cellulose) was supplied by JRS Pharma (Rosenberg, Germany). Lactose monohydrate was purchased from Daejung Chemical. Co., Ltd. (Gyeonggi-do, Korea). Magnesium stearate was purchased from FACI (Genoa, Italy). HPC was purchased from Nippon Soda Co., Ltd. (Tokyo, Japan). L-HPC were supplied by Shin-Etsu Chemical Co., Ltd. (Tokyo, Japan). Starch^®^ 1500 (partially pregelatinized maize starch) was purchased from Colorcon Asia Pacific PTE. Ltd. (Gyeonggi-do, Korea). CS and SSG were purchased from DFE Pharma (Nörten-Hardenberg, Germany). High-performance liquid chromatography (HPLC)-grade acetonitrile and methanol were purchased from JT Baker (Phillipsburg, NJ, USA). All other chemicals used were of analytical grade.

### Animals

Male Sprague–Dawley rats (200–250 g, 7–9 weeks old) were purchased from Orient Bio (Gyeonggi-do, Korea). The rats underwent a period of fasting for approximately 12–18 h prior to drug treatment, with free access to water. All animal experiments were performed in accordance with the National Institute of Health (NIH) guidelines, “Principles of laboratory animal care”, and were approved by the Institutional Animal Care and Use Committee of Chung-Ang University, Seoul, Korea.

### Preparation of SuSMEDDS formulation

Based on our earlier report [1], a drug-free SuSMEDDS formulation was prepared by mixing oil (Capmul^®^ MCM 13.2 mg), surfactant (Tween^®^ 80 59.2 mg), cosurfactant (Transcutol^®^ P 59.2 mg), and a supersaturating agent (Poloxamer 407 13.2 mg). The mixture was vortexed to result in a clear homogenous solution. The VST-loaded SuSMEDDS formulation was obtained by adding 80 mg VST to the drug-free SuSMEDDS (144.8 mg).

### Screening of solid carriers

To solidify the VST-loaded SuSMEDDS, different quantities of various solid carriers were used: Sylysia^®^ 350, Neusilin^®^ US2, and Florite^®^ PS-10 as silica-based adsorbents; HPC, Vivapur^®^ 105, and L-HPC as cellulose-based diluents; and lactose monohydrate, Starch^®^ 1500, and maltodextrin as saccharide-based diluents (Table 1). Solidifying behavior was determined using the levigation method as reported previously [9, 39, 40]. Solid carriers were incrementally added and blended with a fixed quantity of SuSMEDDS in a mortar, and blending was discontinued prior to the formation of a non-flowing cohesive mass. Physical properties of the solidified mass were evaluated for flow characteristics, droplet size on reconstitution, and drug contents and dissolution.

#### Determination of flow properties

Flow behavior of the solidified mass was evaluated using CI, calculated using the equation: CI = [ρ(tapped) - ρ(bulk)]·100/ρ(tapped), where ρ(bulk) and ρ(tapped) are the bulk and tapped densities, respectively [41]. Apparent bulk and tapped bulk densities were measured using the cylinder method, with a powder tester (ABD-100, Tsutsui Scientific Instruments Co. Ltd., Tokyo, Japan). Accurately weighed granule samples were poured into a cylinder and the volume was measured to obtain the apparent bulk density; separately, a sample was tapped 100 times to measure tapped bulk density. In addition, CSR (g/mL) was obtained, defined by the minimum mass (g) of solid carrier required to solidify the unit volume of SuSMEDDS, resulting in a critical constant value of CI

#### Reconstitution study

Solidified mass (equivalent to 80 mg of VST) was dispersed in 250 mL of distilled water (DW). The mixture was gently vortexed (EYELA, Cute Mixer CM-1000, Tokyo, Japan) and centrifuged (Micro 17TP; Hanil Science, Incheon, Korea) at 16,000 *g* for 10 min to remove the water-insoluble solids. The size of dispersed droplets in the supernatant was determined using a photon correlation spectrometer (Zetasizer Nano-ZS, Malvern Instrument, Worcestershire, UK).

#### Drug content determination

Solidified mass (100 mg) was dispersed in methanol (10 mL), mixed thoroughly, and sonicated for 30 min with a bath type sonicator (Model 2210, Branson Ultrasonics Co., Danbury, CT, USA) to extract VST. Samples were filtered through a membrane filter (0.45 μm, PVDF, Smartpor^®^), and the filtrate was appropriately diluted with methanol for HPLC analysis. VST concentration was computed from the validated calibration curve of the drug in methanol (100%, v/v), and drug contents were expressed as percentages of the theoretical quantity.

### HPLC analysis of VST

VST concentration was determined using HPLC. The HPLC system included a pump (W2690/5; Waters Corporation, Milford, MA, USA), ultraviolet detector (W2489; Waters Corporation), data station (Empower 3; Waters Corporation), and chromatographic C18 column (250 × 4.6 mm, 5 μm; Shiseido, Tokyo, Japan), maintaining a flow rate of 1.0 mL per minute at 25 °C. The isocratic mobile phase was composed of acetonitrile and distilled water (60:40 [v/v]). The pH was adjusted to 3.0 using 10% phosphoric acid. Finally, 20 μL of each sample was injected into the column, and the absorbance was measured with ultraviolet detection at 247 nm.

### *In vitro* dissolution test

*In vitro* dissolution tests were performed using the USP apparatus II (paddle) method with a Vision^®^ Classic 6™ Dissolution Tester and Vision^®^ heater (Hanson Research, Chatsworth, CA, USA). A pH 1.2 medium was prepared by dissolving 2 g of sodium chloride in 7 mL of hydrochloric acid and diluting with DW to 1000 mL. Each formulation, containing 80 mg of VST, was introduced into the pH 1.2 dissolution medium (500 mL) at 37 ± 0.5°C, and stirred at 100 rpm. Samples (5 mL) were taken at predetermined time points (5, 15, 30, 60, 90, and 120 min) and filtered through a 0.45-μm polyvinylidene difluoride membrane. After appropriate dilution of the filtrate with methanol, the VST concentration in each sample was assayed using HPLC as described above. For comparison of dissolution profiles, DE was calculated using the trapezoidal rule as follows:

$$\text{DE}\left(\%\right)=\left[\int_{\text{t}1}^{\text{t}2}\text{y dt}/{\text{y}}_{100}\left(\text{t}2-\text{t}1\right)\right]\times 100$$

where y is the percentage of dissolved product [42].

### Preparation and characterization of S-SuSMEDDS granules

Based on the screening results, two solid carriers (Florite^®^ PS-10 and Vivapur^®^ 105) were selected and used in the solidification process at different ratios by blending with SuSMEDDS as described above. The final mixture, a free-flowing powder with a non-greasy appearance, was passed through a 500-μm sieve, and the resultant S-SuSMEDDS granules were stored at ambient temperature in an airtight container.

#### Experimental design

D-optimal mixture design was used to optimize the ratio of Florite^®^ PS-10 and Vivapur^®^ 105 for preparing the S-SuSMEDDS granules. Design-Expert software version 7 (Stat-Ease Inc, Minneapolis, USA) was used for developing and evaluating experimental design. The experiment was designed using the two components as independent variables: Florite^®^ PS-10 (X_1_) and Vivapur^®^ 105 (X_2_) were set within ranges of 0–100%. CSR (Y_1_) and D_15_ (Y_2_) were evaluated as response variables to determine the optimal S-SuSMEDDS granule formulation, for maximal drug release with minimal quantity.

#### Solid-state characterization

Solid-state properties of the drug powder, solid carriers, and optimized S-SuSMEDDS granules were investigated using SEM and DSC. Morphological features of VST powder, Florite^®^ PS-10, Vivapur^®^ 105, and VST-loaded S-SuSMEDDS granules were investigated using a scanning electron microscope (S-3400N, Hitachi, Tokyo, Japan): samples were fixed on a metal plate, sputtered for 90 s with platinum (IonSputter, E-1010, Hitachi, Tokyo, Japan), and photographed at an excitation voltage of 5 kV. DSC measurements were performed using a DSC-Q20 (TA instrument, New Castle, DE, USA): samples (2–5 mg) were placed in an aluminum pan, and measurements were taken over a temperature range of 30–300 °C, at a heat rate of 5 °C/min under nitrogen flow (20 mL/min).

### Preparation and evaluation of S-SuSMEDDS tablets

S-SuSMEDDS granules were blended with selected superdisintegrants (CS, KC, and SSG) and lubricant (magnesium stearate) using a cube mixer (Type AR400ES, Erweka^®^ GmbH, Heusenstamm, Germany) for 5 min. The blended mixture was directly compressed into tablets on a single-punch tablet machine (HANDTAB-200, Ichihashi-Seiki Co. Ltd., Kyoto, Japan) at a compression force of 500–2000 kgf using 12 mm standard circular concave punches, and the resultant S-SuSMEDDS tablets were stored at ambient temperature in an airtight container.

#### Experimental design

3-LFD was used to optimize the conditions for S-SuSMEDDS tablet preparation. The experiment was designed using three components as independent variables: the compression force (X_1_) and concentration of superdisintegrant (wt% of S-SuSMEDDS granules; X_2_) were used as numerical factors, and were set within ranges of 500–2000 kgf and 2–8%, respectively; the type of superdisintegrant (X_3_) was used as a categorical factor, and included CS, KC, and SSG. Tablet hardness (Y_1_), Disint% (Y_2_), DE (Y_3_), and the total mass of S-SuSMEDDS tablet (Y_4_) were evaluated as the response variables, to determine the optimal formulation for desirable physiochemical characteristics.

#### Evaluation of physical strength

Physical testing of optimized S-SuSMEDDS tablets was performed after a relaxation period of at least 24 h. Weight-variation tests were performed with 20 individually weighed tablets using a balance (XS603S analytical balance; Mettler-Toledo, Columbus, OH, USA). The thickness and diameter of ten tablets were measured individually using Vernier calipers (CD-15APX; Mitutoyo, Kawasaki, Japan). Tablet friability was calculated as the percentage of weight loss (4 min, 25 rpm, 20 tablets) using a friability tester (PTF 20E; Pharma Test, Hainburg, Germany). A hardness tester (Smart-Test 50; Pharmatron, Solothun, Switzerland) was used to determine tablet hardness. Ten tablets from each formulation were tested.

#### Disintegration test

Disintegration tests were performed in 900 mL distilled water at 37°C for 30 min using a disintegration tester (DIT-200, Gyeonggi-do, Korea). After the disintegration test, the remaining sample was taken and dried in an oven at 40°C for 24 h to determine Disint%, calculated by 100^*^(W_i_-W_f_)/W_i_, where W_i_ and W_f_ are the masses (mg) of samples before and after the disintegration test, respectively.

### *In vivo* oral absorption study

#### Oral administration and plasma sampling

After rats had undergone overnight fasting (12–18 h), VST treatments were administered using a Torpac^®^ Kit. Test subjects were randomly divided into three groups (n = 5–7): Group 1 received Diovan^®^ (commercial product, ground into powder), Group 2 received SuSMEDDS, Group 3 received optimized S-SuSMEDDS granules. In all treatments, a dose equivalent to 10 mg/kg VST was accurately weighed and dispensed into hard gelatin capsules (Torpac capsule size 9) (Torpac, Fairfield, NJ, USA) by means of a stand, funnel, and tamper (Torpac kit, Torpac). All capsules were administered directly into the stomach by using a dosing syringe plunger (Torpac kit, Torpac). Blood samples (approximately 0.3 mL) were collected from the retro-orbital plexus into heparinized tubes at predetermined time points (20 and 40 min, 1, 1.5, 2, 4, 7, 12, and 24 h) and were centrifuged at 16,000 *g* for 15 min. Plasma samples were stored at -80°C until analysis by liquid chromatography-tandem mass spectrometry (LC-MS/MS).

Whole plasma samples (50 μL) were mixed with 700 μL of methanol and 20 μL of internal standard (IS) solution (10,000 ng/mL VST-d3 in 50% methanol) and were vortexed for 3 min. After centrifugation at 16,000 *g* for 5 min, 20 μL of the supernatant was carefully transferred to a test tube and was evaporated using nitrogen. The dry residue was reconstituted in 480 μL of DW, and the mixture was vortexed and centrifuged at 16,000 *g* for 5 min. Finally, 100 μL of the supernatant was transferred to autosampling vials for introduction into the LC-MS/MS system.

#### Determination of VST in plasma samples using LC-MS/MS

Liquid chromatographic separation was performed using an Agilent 1260 autosampler (Agilent Technologies Inc, Santa Clara, CA, USA). The temperature of the autosampler was maintained at 7°C, and 5 μL of each reconstituted sample was separated into components using a Waters Atlantis dC18 column (50 × 2.1 mm, 3 μm; Milford, MA, USA) at 35°C. An isocratic mobile phase was used, containing 10 mM ammonium formate (pH 2.7) and methanol (20:80, [v/v]), at a flow rate of 0.3 mL per min.

The components eluted from the column were delivered into an API 4500 triple quadrupole mass spectrometer (Applied Biosystems/MDS SCIEX, Foster City, CA, USA) with electrospray ionization in positive ion mode for ion production. The ion spray voltage was set at 5.5 kV, and the source temperature was set at 550°C. Multiple reaction monitoring was performed using nitrogen as the collision gas. Analytes were detected by monitoring the transitions 436.2 (Q1) → 291.0 (Q3) and 439.2 (Q1) → 294.0 (Q3) m/z, with a declustering potential of 28 V and collision energies of 23 V, for VST and IS, respectively. Nebulizer gas (Gas 1) at 40°C and heater gas (Gas 2) temperatures were both set at 70°C. For quantifying VST in the plasma samples, each peak area of VST was divided by that of the internal standard, and the ratio was compared with a calibration curve obtained using VST standard solution in the same manner.

#### PK assessment

Data analysis was performed using the BA Calc 2007 pharmacokinetic analysis program (Ministry of Food and Drug Safety [formerly Korea Food and Drug Administration], Chungcheongbuk-do, Korea). The AUC from 0 to 24 h was calculated using the linear trapezoidal rule. The C_max_ and T_max_ were determined directly from the concentration-time data. RBA was calculated by dividing the AUCs of the test samples with those of the VST suspension.

### Statistical analysis

All data are expressed as mean ± standard deviation (SD). Statistical significance was determined using Student’s *t*-test, with *p* < 0.05 considered statistically significant. Design-Expert software was used to determine the simultaneously assigned statistical values of all responses.

## CONCLUSIONS

Novel formulations of VST-loaded S-SuSMEDDS granules and tablets were successfully developed using D-optimal mixture design and 3-LFD, respectively, resulting in percentage prediction errors of <10%. SuSMEDDS composed of VST, Capmul^®^ MCM, Tween^®^ 80, Transcutol^®^ P, and Poloxamer 407 was efficiently solidified with Florite^®^ PS-10 and Vivapur^®^ 105, and the resultant granules showed good flow properties and rapid drug dissolution. By introducing CS as a superdisintegrant, S-SuSMEDDS tablets were successfully formulated, resulting in fast disintegration and high dissolution efficiency. In PK studies in rats, the RBA of the optimized granules was 107% and 222% of the values obtained for SuSMEDDS and Diovan^®^ powder, respectively. Therefore, we suggest that the optimized S-SuSMEDDS formulations offer great potential for developing solid dosage forms, with improved oral absorption of poorly water-soluble drugs such as VST.

## Abbreviations

3-LFD: 3-level factorial design
AUC: Area under the curve
RBA: Relative bioavailability
CI: Carr’s index
C_max_: Maximum plasma concentration
CS: Croscarmellose sodium
CSR: Critical solidifying ratio
D_15_: Percentage of drug released in 15 min
DE: Dissolution efficiency
Disint%: Percentage of disintegration in 30 min
DSC: Differential scanning calorimetry
DW: Distilled water
GI: gastric intestinal
HPC: Hydroxypropyl cellulose L type
HPLC: High-performance liquid chromatography
KC: Kollidon^®^ CL
LC-MS/MS: Liquid chromatography-tandem mass spectrometry
L-HPC: Low-substituted hydroxypropyl cellulose B1
PK: Pharmacokinetic
R^2^: Squared correlation coefficient
RBA: Relate bioavailability
SEM: Scanning electron microscopy
SMEDDS: Self-microemulsifying drug delivery system
SSG: Sodium starch glycolate
S-SuSMEDDS: Solidified supersaturable self-microemulsifying drug delivery system
SuSMEDDS: Supersaturable self-microemulsifying drug delivery system
T_max_: Time to reach the maximum plasma concentration
VST: Valsartan
